# Targeting Atp6v1c1 Prevents Inflammation and Bone Erosion Caused by Periodontitis and Reveals Its Critical Function in Osteoimmunology

**DOI:** 10.1371/journal.pone.0134903

**Published:** 2015-08-14

**Authors:** Sheng Li, Liang Hao, Lin Wang, Yun Lu, Qian Li, Zheng Zhu, Jian-Zhong Shao, Wei Chen

**Affiliations:** 1 Department of Pathology, University of Alabama at Birmingham, Birmingham, AL, 35294, United States of America; 2 College of Stomatology, Nanjing Medical University, Nanjing, 210029, People’s Republic of China; 3 Life Science College, Zhejiang University, 388 Yuhang Road, Hangzhou, 310058, People's Republic of China; University of Toronto, CANADA

## Abstract

Periodontal disease (Periodontitis) is a serious disease that affects a majority of adult Americans and is associated with other systemic diseases, including diabetes, rheumatoid arthritis, and other inflammatory diseases. While great efforts have been devoted toward understanding the pathogenesis of periodontitis, there remains a pressing need for developing potent therapeutic strategies for targeting this pervasive and destructive disease. In this study, we utilized novel adeno-associated virus (AAV)-mediated Atp6v1c1 knockdown gene therapy to treat bone erosion and inflammatory caused by periodontitis in mouse model. Atp6v1c1 is a subunit of the V-ATPase complex and regulator of the assembly of the V0 and V1 domains of the V-ATPase complex. We demonstrated previously that Atp6v1c1 has an essential function in osteoclast mediated bone resorption. We hypothesized that Atp6v1c1 may be an ideal target to prevent the bone erosion and inflammation caused by periodontitis. To test the hypothesis, we employed AAV RNAi knockdown of Atp6v1c1 gene expression to prevent bone erosion and gingival inflammation simultaneously. We found that lesion-specific injection of AAV-shRNA-Atp6v1c1 into the periodontal disease lesions protected against bone erosion (>85%) and gingival inflammation caused by *P*. *gingivalis W50* infection. AAV-mediated Atp6v1c1 knockdown dramatically reduced osteoclast numbers and inhibited the infiltration of dendritic cells and macrophages in the bacteria-induced inflammatory lesions in periodontitis. Silencing of Atp6v1c1 expression also prevented the expressions of osteoclast-related genes and pro-inflammatory cytokine genes. Our data suggests that AAV-shRNA-Atp6v1c1 treatment can significantly attenuate the bone erosion and inflammation caused by periodontitis, indicating the dual function of AAV-shRNA-Atp6v1c1 as an inhibitor of bone erosion mediated by osteoclasts, and as an inhibitor of inflammation through down-regulation of pro-inflammatory cytokine expression. This study demonstrated that Atp6v1c1 RNAi knockdown gene therapy mediated by AAV-shRNA-Atp6v1c1 is a promising novel therapeutic approach for the treatment of bone erosion and inflammatory related diseases, such as periodontitis and rheumatoid arthritis.

## Introduction

The ultimate outcome of periodontitis is alveolar bone and tooth loss, which results from the interaction between oral biofilm microorganisms and the host immune response in the periodontitis lesion area. Different cell types have been demonstrated to participate in this inflammatory progress, such as T lymphocytes, macrophages, and dendritic cells [1, 2]. Besides oral disease, periodontal diseases are associated with many systemic diseases such as rheumatoid arthritis, diabetes, infective endocarditis, and scorbutus (scurvy) [3, 4]. This is due to the fact that immune cells in the lesion areas activate nuclear factor kappa-B ligand (RANKL), lead to osteoclast activation [5], and result in bone and tooth loss in a state of inflammation [6]. Previous studies revealed that osteoclasts induced in this inflammatory response are the leading cause of tooth and alveolar bone loss [7]. During the process of inflammation, a multi-unit vacuolar-type H^+^-ATPase (V-ATPase) complex decreases the pH at the bone surface. This extracellular acidification was induced by inflammatory cytokines, followed by the activated osteoclasts’ resorption of bone around the root or teeth. The decrease of pH is important for bone erosion related to osteoclasts [8, 9].

The *ATP6V1C1* gene encodes V-type proton ATPase subunit C1 [10, 11]. This gene is responsible for encoding the enzyme vacuolar ATP (V-ATP enzyme) and acidifying components within the multi-subunit enzyme-mediated eukaryotic cellular compartments. V-ATPase-dependent acidification is an important step for intracellular processes, including zymogen activation, receptor-mediated endocytosis, and synaptic vesicle proton gradient generation [12, 13]. Our previous research determined that Atp6v1c1 is mainly expressed in osteoclasts, whereas subunits Atp6v1c2a (C2a) and Atp6v1c2b (C2b) are not [9]. C1 expression is highly induced by RANKL during the process of osteoclast differentiation. C1 interacts with Atp6v0a3 (a3), and is mainly localized on the ruffled border of activated osteoclasts [9]. A previous study showed that in addition to being an essential component of V-ATPases, Atp6v1c1 may regulate filament actin arrangement in breast cancer cells [14]. Silencing of Atp6v1c1 prevents breast cancer growth and bone metastasis [15], indicating the potential multiple functions of Atp6v1c1 in normal cell functions and diseases. Since cancer growth and metastasis are related to immune response, we hypothesize that inhibition of Atp6v1c1 may also prevent the immune response and the following bone erosion. As a subunit of Atp6i that is expressed in both osteoclasts and immune cells such as macrophages and dendritic cells [9, 16], Atp6v1c1 should have an osteoimmune function during the development of periodontitis.

In our current study, we chose the adeno-associated virus (AAV) as the viral vector, which has been shown to be useful in humans [17, 18]. AAV has been verified as safe and effective, has good compatibility and is accompanied by a minor immune response [19, 20]. Recent studies have already indicated AAV’s ability to function long-term in different doses [21], which is promising for gene therapy *in vivo* [19]. Therefore, we used RNAi introduced with AAV to explore the possible effect of Atp6v1c1 silencing [22]. In this study, our data indicate that AAV-shRNA-Atp6v1c1 treatment can significantly attenuate the bone erosion and inflammation caused by periodontitis and suggest that Atp6v1c1 RNAi knockdown gene therapy could be a novel therapeutic approach for the treatment of periodontitis.

## Materials and Methods

For complete Materials and Methods, please see S1 File.

### Animals

Adult (8 wks) female wild-type (WT) BALB/cJ mice (from Jackson Laboratory) were used for current study [9, 23, 24]. All of the mice were divided into 3 groups: (1) Normal group (mice without bacteria) (n = 7); (2) Bacteria infection treated with AAV-shRNA-Atp6v1c1 (AAV-sh-Atp6v1c1) (n = 7); (3) Bacteria infection treated with AAV-sh-luc-YFP (n = 7). The experiments were performed three times on three different occasions, generating a total sample number 21 for each group. All mice were maintained under a 12-hour light–dark cycle with ad libitum access to regular food and water at the University of Alabama at Birmingham (UAB) Animal Facility. The Protocol used in our current experiment was approved by the Institutional Animal Care and Use Committee of UAB (Animal Protocol Number 131209236).

### Infection with *Porphyromonas gingivalis* strains

*Porphyromonas gingivalis* W50 (ATCC: 53978) were seeded and cultured on sheep’s blood agar plates supplemented with hemin and vitamin K (BAPHK) for 3 days in an oxygen-free environment. Next a single clone was harvested and transferred to Trypticase Soy Broth supplemented with hemin and vitamin K. Bacteria were harvested on the 4^th^ day and centrifuged for 30min and then resuspended. The bacteria concentrations of each species were determined via optical density readings at 600nm (One OD unit equals 6.67*10^8^ bacteria). The bacteria density of each species was adjusted to 10^10^cells/ml in PBS containing 2% carboxymethylcellulose (CMC: Sigma-Aldrich). The procedure of periodontal infection was conducted following the previously described protocol with some modifications [25]. In brief, all animals received antibiotic treatment for 3 days to reduce the original oral confounders, followed by 3 days without antibiotic, prior to oral inoculation with 0.2ml *P*. *gingivalis* in CMC in 20 μl with a dental micro-brush once per day for 4 consecutive days. To verify bacterial effectiveness, the sample from the mice oral cavity was taken on day 14 after infection. The sterile cotton swab was used to collect the bacteria plaque and samples were incubated anaerobically to identify *P*. *gingivalis* [25].

### AAV-shRNA-Atp6v1c1 transduction of *P*. *gingivalis* W50 infected mice

In the present study, we injected AAV-sh-Atp6v1c1 in specific sites as described previously [19]. On the 4^th^ day after initial infection, the mice were anesthetized via peritoneal injection with 62.5mg/kg ketamine and 12.5mg/kg xylazine. Next, AAV-sh-Atp6v1c1 was injected approximately 0.3–0.5mm above the gingival margin of the maxillary molars on the right and left palatal aspects with 3ul containing 2 x10^9^ packaged genomic particles in either AAV-sh-Atp6v1c1 or AAV-sh-luc-YFP viral vector using 5-μl Hamilton syringe attached to a microinfusion pump (World Precision Instruments, Sarasota, FL). The injection was repeated every day for 7 consecutive days. In the negative control group (normal), mice were maintained without any treatment.

### Sample harvest and preparation

8 weeks after initial infection, all animals were sacrificed by CO_2_ inhalation and the maxillae were harvested. For measurements of bone height, samples from the left side were defleshed thoroughly in 2.6% sodium hypochlorite for 30–40 minutes [26], rinsed in tap water three times, soaked in 70% alcohol, and then stained with 1% methylene blue, and observed by microscope for bone loss measurements. For histological analysis, the samples from the right side were fixed in 4% paraformaldehyde and according to standard protocol. Samples for paraffin sections were fixed in 4% formaldehyde for 24 hours and washed with PBS, decalcified in 10% EDTA in 0.1M TRIS solution (pH = 7.0) for 10 days. After washing with 1XPBS three times, samples were embedded into paraffin after series dehydration. Another three samples from the right side in every independent experiment were obtained for Real-Time quantitative PCR (qRT-PCR) analyses and Enzyme-linked immunosorbent assays (ELISAs). Gingival tissues and/or alveolar bone were isolated under a stereo microscope. Gingival tissues and alveolar bone from three samples were pooled for qRT-PCR, and gingival tissues from another three samples were pooled for ELISAs for cytokines. All of these experiments were repeated three times.

### Bone resorption measurements

The imaging protocol was described previously [27, 28]. The picture of molar teeth, roots and maxillary bone were captured by using Stereomicroscope. The bone loss area was calculated by using image software (Adobe, San Jose, CA, USA). In this procedure, the bone resorption area began at the cemento-enamel junction, including the areas of the root, to the alveolar ridge, which was measured using ImageJ (Bethesda, USA). The results were expressed in mm^2^.

### Histological and immunohistochemical analysis

Hematoxylin & eosin (H&E) staining and calculation were done as described previously [29, 30]. Anti-ATP6v1c1 (H-300) (Santa Cruz, CA), rat monoclonal anti-F4/80 (eBioscience, San Diego, CA) and rabbit anti-mouse CD11c (Santa Cruz, Dallas, Texas, USA) were used for Immunohistochemical analysis (IHC) separately. The IHC analyses in the present study were repeated three times separately.

### Real-time quantitative PCR (qRT-PCR)

The tissues in the lesion area were isolated and stored at -80°C for determination of the effect of Atp6v1c1 on the levels of nucleic acid in inflammatory periodontal tissues as described previously [27, 28, 31, 32]. qRT-PCR was carried out using TaqMan probes (Applied Biosystems, Life Technologies, Grand Island, NY, USA) as listed in S1 Table.

### Enzyme-linked immunosorbent assay (ELISA)

ELISA was applied to determine the effect of Atp6v1c1 on the levels of protein in lesion areas as previously described [28, 32, 33]. The following ELISA kits have been used: IL-1α (Biolegend, San Diego, CA, USA), IL-6 (Biolegend, San Diego, CA, USA), and IL-17A (ebioscience, San Diego, CA). The results were expressed as pg cytokine/mg tissue.

### Statistical analysis

All of the experimental data were presented as mean ± SD. For the parametric data, the two-tailed Student's t-test and one-way ANOVA test were used for analysis. For non-parametric data, Mann-Whitney U test was used for analysis (*P* values <0.05 or *U* values > 1.96).

## Results

### The expression of Atp6v1c1 was knocked down efficiently by AAV-shRNA Atp6v1c1 as well as bone erosion related to osteoclasts in vitro

**Fig 1:**
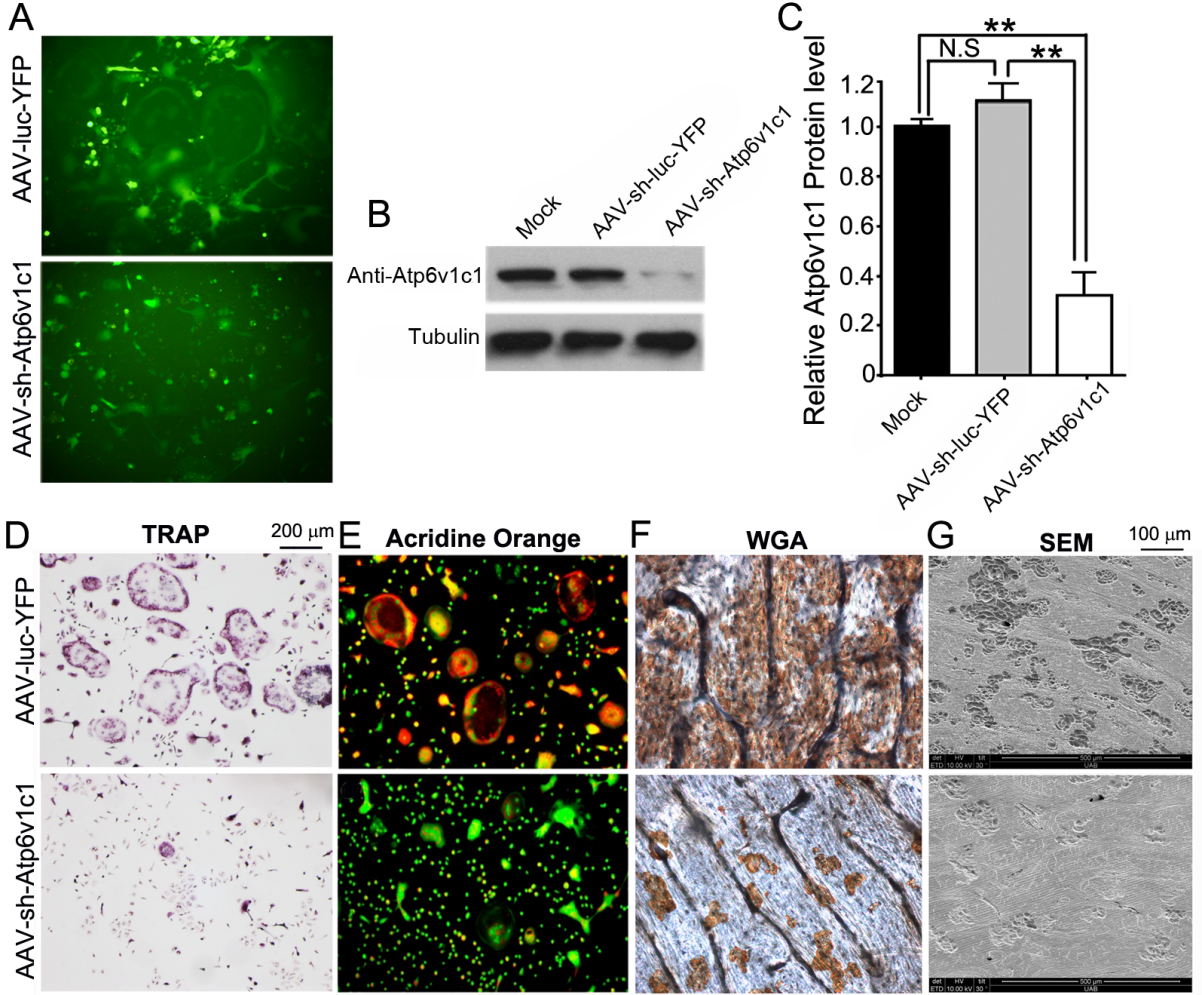
The expression of Atpv1c1 was efficiently knocked down by AAV-sh-*Atp6v1c1*. (A-G) M-CSF/RANKL was applied for 3 days to allow differentiation of osteoclasts, which were then transduced with AAV-sh-Atp6v1c1, AAV-sh-luc-YFP, or untreated (mock). (A) Transduction of osteoclasts was successful indicated by fluorescence. (B, C) Atpv1c1 protein level by Western blot showed that AAV-sh-Atp6v1c1 treated osteoclasts have reduced Atp6v1c1 expression significantly (*P*<0.01). (D) TRAP staining revealed that the number of osteoclasts was decreased in the AAV-sh-Atp6v1c1 treatment group. (E) AAV-sh-luc-YFP (control) or AAV-sh-Atp6v1c1 transduced osteoclasts were stained with acridine orange to show extracellular acidification. (F) Wheat germ agglutinin (WGA) was applied for visualizing resorption lacunae and (G) scanning electron microscopy (SEM) showed that osteoclast-mediated bone resorption was inhibited by ATP6v1c1 knockdown. **: *P*<0.01, N.S: No Significance.

In our current study, shRNA target *Atp6v1c1* was generated. Knockdown of *Atp6v1c1* inhibited inflammation and bone resorption simultaneously. We first examined the expression level of YFP in target cells to determine whether AAV-sh-luc-YFP and AAV-sh-Atp6v1c1 can transduce target cells successfully. Osteoclasts were induced by using M-CSF and RANKL in mouse bone marrow (MBM), which was isolated from WT mice with BALB/cJ background, which were then transduced by AAV-sh-Atp6v1c1 or AAV-sh-luc-YFP. Fluorescence of cultured cells showed that transduction of pre-osteoclasts and osteoclasts with AAV-sh-Atp6v1c1 or AAV-sh-luc-YFP was successful (Fig 1A). Western blot analysis was then applied to confirm transduction, and we found that the expression of Atp6v1c1 in osteoclasts treated with AAV-sh-Atp6v1c1 decreased almost 70% compared to osteoclasts treated with AAV-sh-luc-YFP (Fig 1B and 1C). The results indicated that the application of AAV-sh-Atp6v1c1 can efficiently reduce Atp6v1c1 protein expression *in vitro*. Therefore the function of Atp6v1c1 is indispensable for extracellular acidification mediated by osteoclast in bone resorption. We then performed tartrate-resistant acid phosphatase (TRAP) staining, which showed that TRAP-positive osteoclasts decreased significantly in the AAV-sh-Atp6v1c1 group (Fig 1D). The acridine orange staining also showed that the osteoclasts treated with AAV-sh-Atp6v1c1 had weak extracellular acidification (Fig 1E). We accordingly anticipated that knockdown of Atp6v1c1 would inhibit bone resorption as well. Compared to the control group, AAV-mediated knockdown of Atp6v1c1 completely inhibited bone resorption (Fig 1F and 1G). These data clearly showed that AAV-sh-Atp6v1c1 can inhibit acidification and bone resorption mediated by osteoclasts *in vitro*.

### Inhibition of Atp6v1c1 prevents bone erosion in periodontitis lesion areas caused by *P*. *gingivalis* W*50*

**Fig 2:**
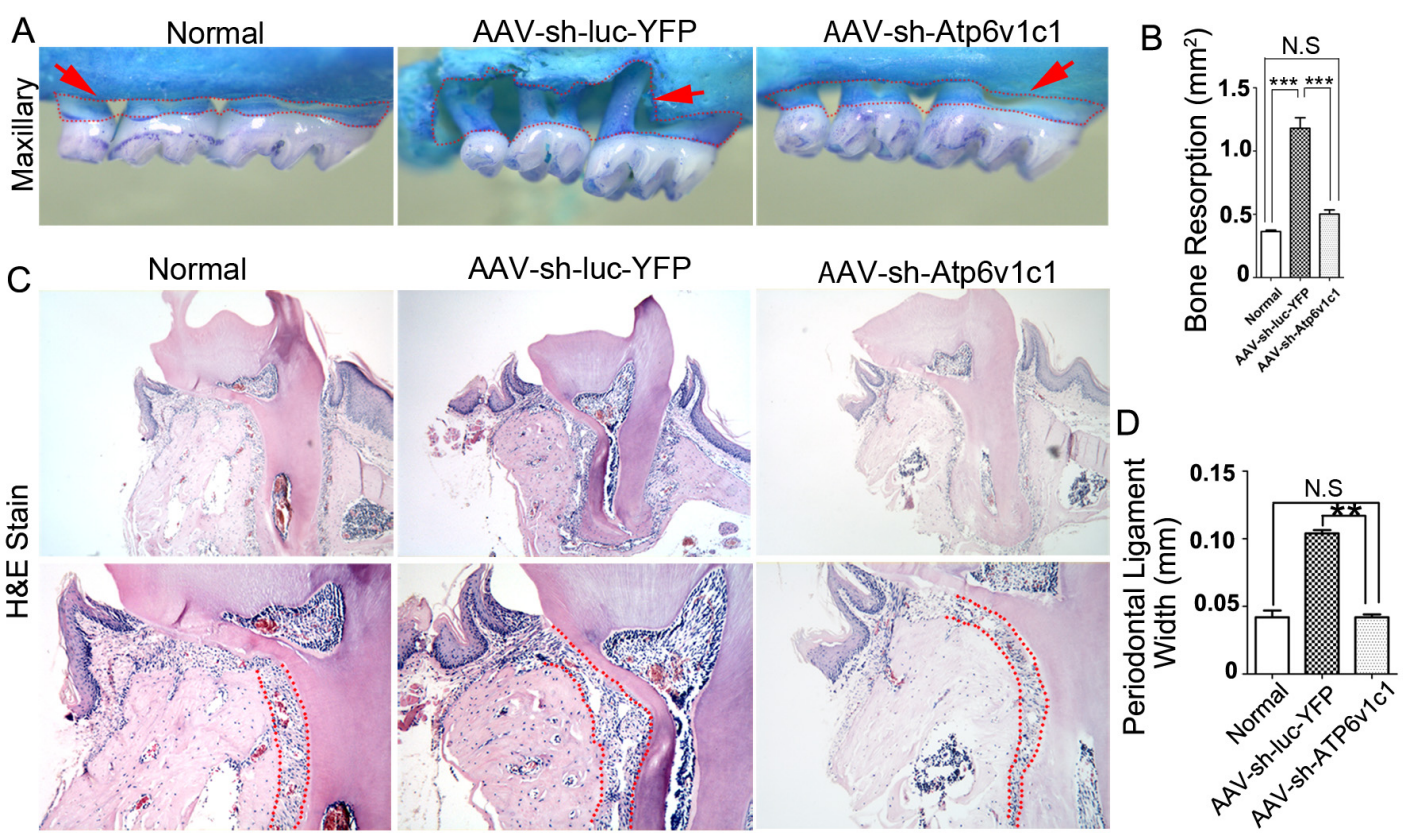
Bone resorption in experimental periodontitis was reduced by Atp6v1c1 knockdown. (A) In the AAV-sh-luc-YFP group with infection, marked bone resorption can be seen and all teeth roots are exposed near the tip of the root in the maxillary (red dotted area). (B) There is almost twice the amount of bone resorption in the AAV-sh-luc-YFP group when compared to the AAV-sh-Atp6v1c1 group. (C) The red dot area shows a widened periodontal ligament area in the AAV-sh-luc-YFP treated disease group. (D) The periodontal ligament width is almost doubled in the AAV-sh-luc-YFP treatment group compared to the AAV-sh-Atp6v1c1 treatment group, indicating that alveolar bone loss was prevented by AAV-sh-Atp6v1c1. **: *P*<0.01, ***: *P*<0.001, N.S: No Significance.

It has been indicated that the *Porphyromonas gingivalis W50* (*P*. *gingivalis W50*) strains can cause severe bone loss in experimental animal models of periodontal disease [27]. We first verified bone resorption induced by this bacteria strain. The results showed that significant bone loss can be caused by *P*. *gingivalis W50* compared to a normal group (*P*<0.05) (Fig 2A and 2B). There was also a significant difference in bone resorption between bacteria infected mice treated with AAV-sh-Atp6v1c1 and with AAV-sh-luc-YFP (Fig 2B). Alternatively, the difference in bone resorption between the normal group and the AAV-sh-Atp6v1c1 treated group was not significant (Fig 2B). The AAV-sh-luc-YFP group infected by bacteria had much more bone loss when compared to the group treated with AAV-sh-Atp6v1c1 (*p*<0.0001) and the normal group (p<0.0001) (Fig 2B). These results indicate that *P*. *gingivalis* W50-stimulated bone erosion can be inhibited by local application of AAV-sh-Atp6v1c1 in the experimental periodontal disease mouse model. The periodontal ligament width between the group with AAV-sh-Atp6v1c1 treatment, normal group and the AAV-sh-luc-YFP group was then examined by H&E sections. Results showed that the periodontal ligament width is significantly higher in the AAV-sh-luc-YFP treated group compared to the normal and AAV-sh-Atp6v1c1 treated group (Fig 2C and 2D), indicating that AAV-sh-Atp6v1c1 prevents the periodontal ligament from damage and reduces alveolar bone resorption.

### AAV-mediated Atp6v1c1 knockdown decreased TRAP positive osteoclasts as well as immune cells in the periodontitis lesion area

**Fig 3:**
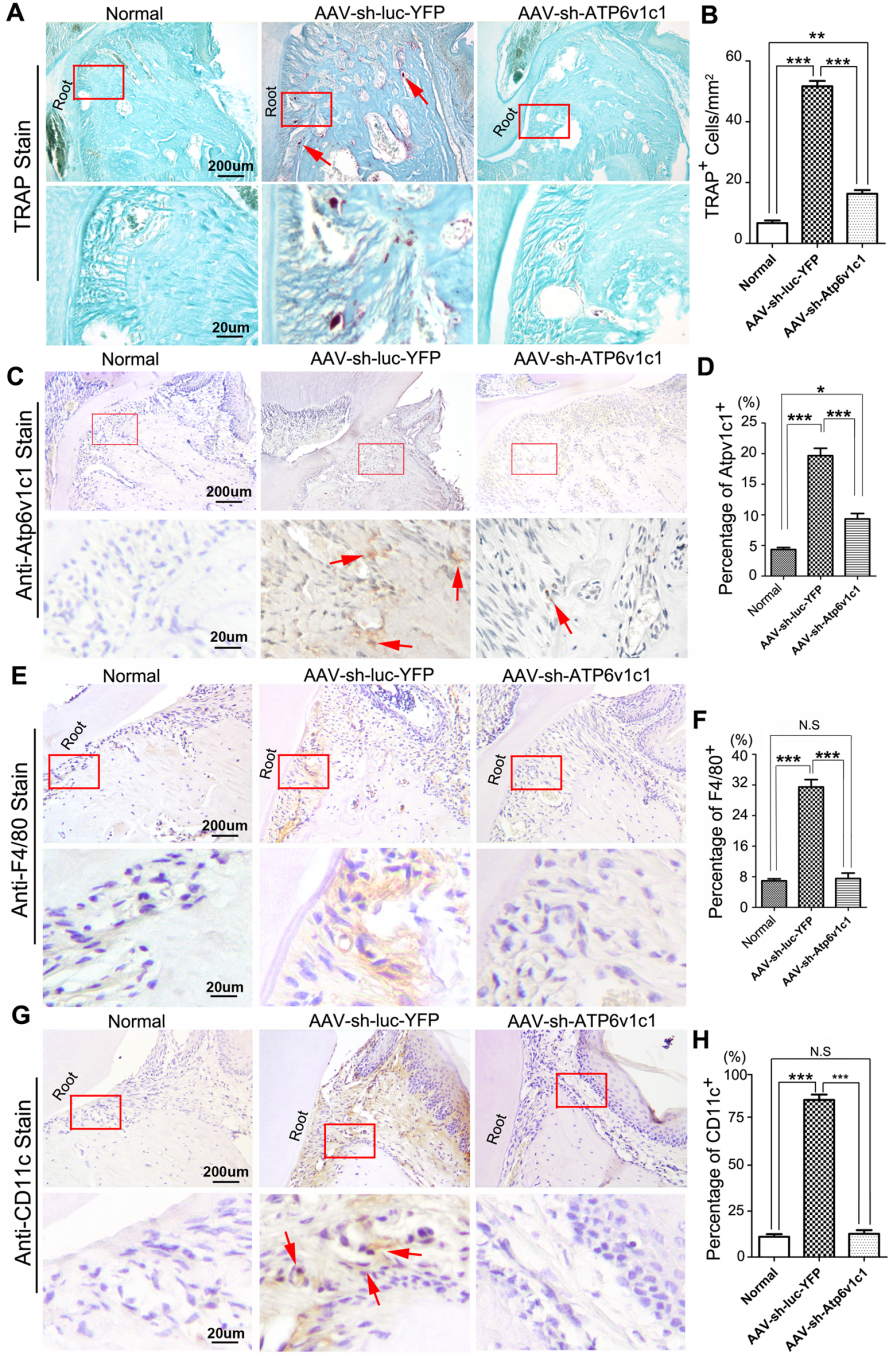
AAV-mediated Atp6v1c1 knockdown decreased TRAP positive cells and Atp6v1c1 expression in the periodontitis lesion area. (A) TRAP staining was used to indicate the decrease in Atp6v1c1 expression in periodontal tissues after AAV-sh-Atp6v1c1 knockdown. The number of osteoclasts decreased significantly (Red arrows). Mature osteoclasts could be found frequently under microscope with higher magnification. (B) Statistical analysis indicates that in the AAV-sh-Atp6v1c1 treatment group, the number of osteoclasts was reduced significantly. (C) Immunohistochemical staining was used to verify the effectiveness of AAV-sh-Atp6v1c1 knockdown in periodontal tissues (Red arrows). (D) Statistical analysis indicates that in the AAV-sh-Atp6v1c1 treatment group, the number of Atp6v1c1 positive cells was reduced significantly. (E) Immunohistochemical staining of F4/80 was applied to the different groups in the periodontitis lesion area. (F) Statistical analysis indicates that the number of F4/80 positive cells was reduced significantly in the AAV-sh-Atp6v1c1 treatment group. (G) Immunohistochemical staining of CD11c was applied to the different groups in the periodontitis lesion area (Red arrows). (H) Statistical analysis indicates that the number of CD11c positive cells was reduced significantly in the AAV-sh-Atp6v1c1 treatment group.*: *P* < 0.05, **: *P* < 0.01, ***: *P*<0.001, N.S: No Significance.

To further explore the possible effect of AAV-sh-Atp6v1c1 on activation of osteoclasts in the lesion area, TRAP staining was applied to histological sections to detect osteoclasts (Fig 3A and 3B). The TRAP positive osteoclasts in the lesion area (periodontal ligament area) were reduced in the AAV-sh-Atp6v1c1 treated group significantly (Fig 3A and 3B) when compared to the group with AAV-sh-luc-YFP treatment (Fig 3B). We then confirmed the expression of Atp6v1c1 in osteoclasts *in vivo*, which is also a confirmation of whether AAV-sh-Atp6v1c1 successfully transduced in the local lesion area. Tooth root sections from bacteria infected mice treated with AAV-sh-Atp6v1c1 or AAV-sh-luc-YFP were applied to IHC using anti-Atp6v1c1. It was found that AAV-sh-Atp6v1c1 reduced expression of Atp6v1c1 *in vivo* notably (Fig 3C and 3D). Further IHC staining of F4/80 and CD11c in different groups showed that AAV-sh-Atp6v1c1 decreased the number of immune cells such as macrophages and dendritic cells (Fig 3E–3H).

### The expression of osteoclast marker genes and cytokines were prevented by AAV-sh-Atp6v1c1 in the periodontitis lesion area

**Fig 4:**
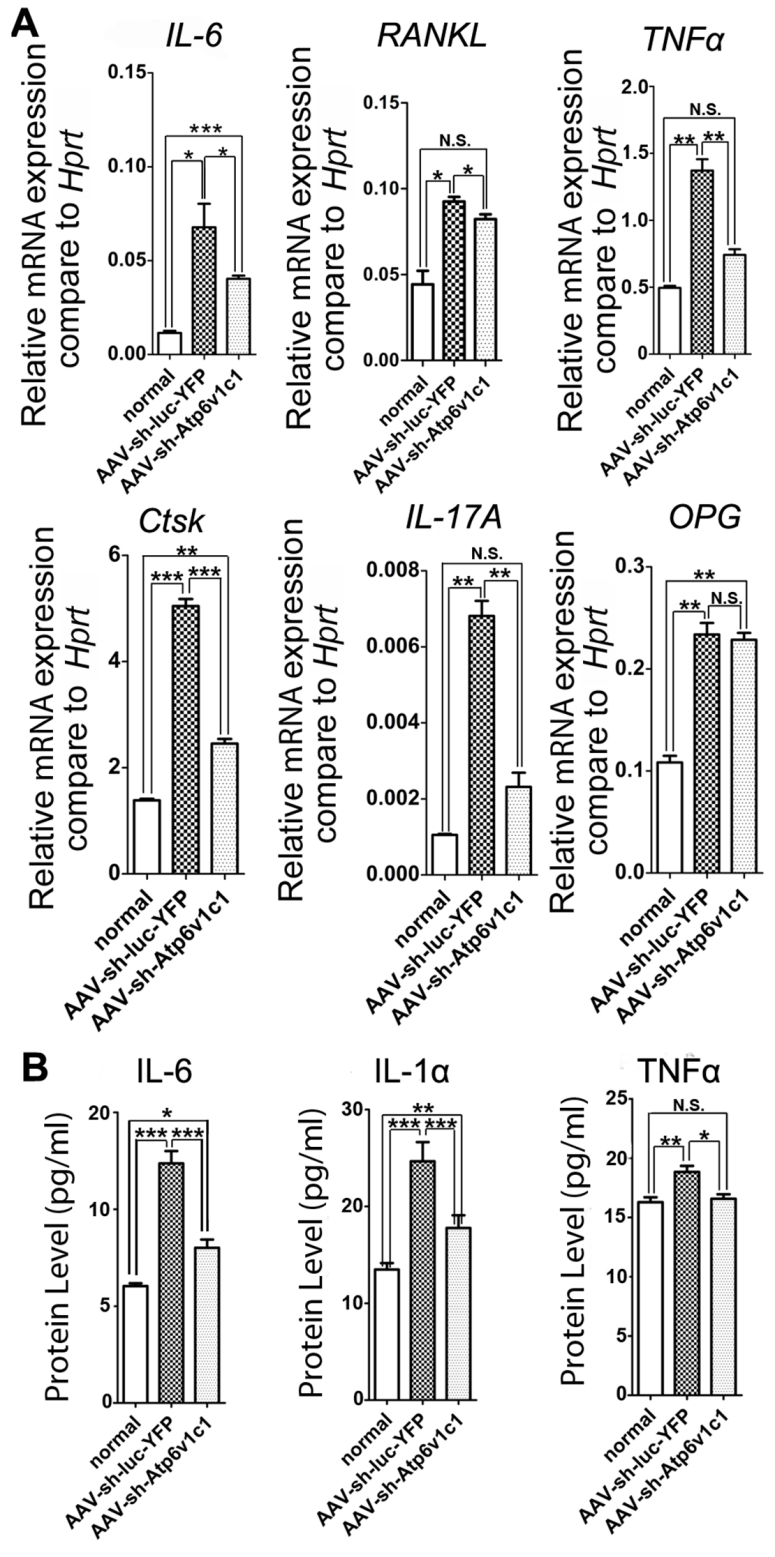
The expression of osteoclast marker genes and cytokines in the periodontal tissues were reduced by AAV-sh-Atp6v1c1. (A) osteoclast-specific genes (*i*.*e*. *Ctsk*), osteoclast differentiation genes (*i*.*e*. *RANKL* and *OPG*), and cytokines (*i*.*e*. *IL-6*, *IL-17A and TNF*α) in different groups. Expression levels were normalized to the housekeeping gene hypoxanthine-guanine phosphoribosyl transferase (*Hprt*). (B) IL-6, IL-1α and TNFα levels in the periodontitis lesion area were detected by ELISA. *: *P* < 0.05, **: *P* < 0.01, ***: *P*<0.001, N.S: *P* > 0.05.

To confirm our *in vivo* histological results, the effect of AAV-sh-Atp6v1c1 on inflammation, ELISA and qRT-PCR was applied to quantify inflammation-related cytokine expression in the periodontitis lesion tissues (Fig 4). Compared to the normal group without infection, we found that some important osteoclast differentiation-related genes [*i*.*e*. *RANKL*, *OPG* and Interleukin-6 (*IL-6*)] and specific osteoclast functional gene [*i*.*e*. Cathepsin K (*Ctsk)*] were increased in the *P*. *gingivalis* W50 infection group treated with AAV-sh-luc-YFP (Fig 4A). Most importantly, AAV-sh-Atp6v1c1 treatment reduced the expression of *RANKL*, *IL*-6 and *Ctsk* in periodontal tissues from the *P*. *gingivalis* W50 group and restored the expression level in the normal group (Fig 4A). Furthermore, the expression of proinflammatory cytokines such as TNFα, IL-6 and IL-1α, which is critical for osteoclast differentiation, were also decreased in the AAV-sh-Atp6v1c1 treated group (Fig 4B). All of these results indicated that Atp6v1c1 knockdown reduces the expression level of genes related to osteoclast and inflammation, and reduces bone erosion and inflammation in periodontitis lesion areas.

## Discussion

Periodontitis is an infectious disease with a bacterial complex pathogen, which has been considered a major cause of periodontitis. In periodontal diseases, the immune response of the host plays an important role [25, 34], by inducing gingival soft tissue damage and osteoclast-mediated bone erosion [35]. In our current study, we found that knockdown of the targeted subunit (Atp6v1c1) of Atp6i by an AAV vector can inhibit inflammation and reduce periodontal disease progression significantly. Our lab also discovered other important genes that are found important in immune response recent years [36–38].

### AAV-sh-Atp6v1c1 is a promising therapeutic agent for periodontitis by inhibiting both osteoclasts and immune cells

Periodontitis has been considered a chronic localized inflammatory disease consisting of the interaction between bacterial pathogens and the host immune response. This subunit is located in the V1 domain of V-ATPase, and is intimately involved in the reversible dissociation of the V1 and V0 domains, and is considered to be directly responsible for regulating the dissociative mechanism of V-ATPase [39, 40]. AAV-sh-Atp6v1c1 inhibited bone destruction *in vitro* through inhibition of acidification mediated by osteoclasts and also reduced pro-inflammatory cytokine expression. Additionally, it reduced the number of osteoclasts and protected mice from bacteria induced bone loss in periodontitis lesion area. These results strongly indicated that application of AAV-sh-Atp6v1c1 gene therapy locally can inhibit alveolar bone loss and tissue damage in periodontitis lesion areas, and may be a promising new strategy for treating periodontal diseases.

### Application of AAV-sh-Atp6v1c1 locally for periodontal disease is effective

Current therapies for periodontal diseases are focused mainly on anti-microbial treatments, which generally have limited application. Gene therapy, on the other hand has great possibilities to achieve an outcome with a sustained therapeutic gene product [35, 41]. In order to achieve a practical therapeutic method that is relatively safe and non-invasive, we should address which genes are best to be studied and which vectors can be employed safely [35]. Our group previously characterized Atp6i [42] and found that it plays important functions in extracellular acidification, which is necessary for immune response and bone resorption. Atp6v1c1 is specific to the proton pump in osteoclasts [8, 9], and the deletion of Atp6v1c1 can result in loss of osteoclast function. It has been shown that AAV2 is relatively safe and effective in different disease models. Another study reported that a relatively long period of sustained expression can be achieved by injection of AAV2 intra-articularly [43]. Osteoprotegerin (OPG) gene transfer can attenuate the osteoclast activity and number in an osteolysis animal model [44]. AAV-IL-4 can also reduce the collagen-induced arthritis (CIA) and inflammatory responses [20]. Sara et al. demonstrated the potential for *in vivo* AAV2-mediated immune gene therapy and provided data on the inter-relationship between tumor vasculature and immune cell recruitment [45, 46]. In our current study, we applied injections of AAV-sh-Atp6v1c1 to treat periodontitis-mediated bone resorption and inflammation. The efficiency of Atp6v1c1 inhibition may reflect the important function of the molecules in the development of periodontitis, in osteoclastic resorption and inflammatory processes that result in tissue damages, as well as in the formation of osteoclasts. Delivery of gene therapy locally is much more efficient and more effective than systemic administration since the synthesis of therapeutic gene products was restricted to the lesion specific area, which can minimize the side effects and the costs of treatment [35]. In our current study, the results showed that AAV-sh-Atp6v1c1 not only prevents inflammation and bone erosion in periodontitis, but it may also be useful for other inflammatory diseases.

Our qRT-PCR and ELISA results also demonstrated that inflammation in the AAV-sh-Atp6v1c1 treatment group decreased significantly. The inflammation reduction in the AAV-sh-Atp6v1c1 treatment group may be due to the down regulation of *IL-6* (a pro-inflammatory cytokine associated with periodontitis). It is reported that IL-6, which is expressed by osteoblasts, promotes inflammation as well as bone resorption [47]. According to the results in the present study, AAV-sh-Atp6v1c1 not only reduced the expression of Atp6v1c1, but also reduced inflammatory cytokine expression. After extraction of protein from tissue samples from different groups, the ELISA assays were performed and the results have been analyzed to find more mechanistic information. The expression of T-cell mediated cytokine IL-6 as well as TNF-α and IL-1α was decreased by application of AAV-sh-Atp6v1c1. In all of the cytokines analyzed by ELISA in our present study, IL-1α is a close relative with bone resorption. Our results indicate that in the *P*. *gingivalis*-infected group treated with AAV-sh-Atp6v1c1, the expression level of IL-1α decreased significantly. Because it has been reported that IL-1α knockdown may inhibit the circuits between inflammatory processes and bone resorption, AAV-sh-Atp6v1c1 treatment can interfere with this positive feedback also and reverse bone resorption in the progression of periodontitis.

Our present findings possess a critical insight into Atp6v1c1 osteoimmune function between bone resorption and inflammatory host responses mediated by bacterial pathogens in periodontitis. These observations highlight a critical pathogenic role for Atp6v1c1 in these settings and identify Atp6v1c1 as a logical target for the suppression of periodontal bone destruction and inflammation.

## Supporting Information

S1 File Supplemental Materials and Methods. (DOCX)

S1 Table qRT-PCR probe Numbers. (DOCX)
