# Supplementary material for: Targeting Atp6v1c1 Prevents Inflammation and Bone Erosion Caused by Periodontitis and Reveals Its Critical Function in Osteoimmunology
Source: PLoS One. 2015 Aug 14;10(8):e0134903. doi: 10.1371/journal.pone.0134903 (PMC4537256; doi:10.1371/journal.pone.0134903)
Supplement: S1 File — (DOCX) [file pone.0134903.s001.docx]

**Supplemental Materials and Methods**

**Cells and Cell Culture**

Pre-osteoclasts and mature osteoclasts in primary culture were generated from mouse bone marrow (MBM) as previously described. Briefly, MBM was obtained from tibiae and femora from six-week-old female WT BALB/cJ mice [1]. MBM cells (1-2×10^5^) were seeded into wells of a 24-well plate and 1×10^6^ MBM seeded into wells of 6-well plate. MBM was cultured in *α*-modified Eagle’s medium (α-MEM; GIBCO-BRL) with 10% fetal bovine serum (FBS; GIBCO-BRL) containing 20 ng/ml macrophage colony-stimulating factor (M-CSF) (R&D Systems). After 1 day, cells were further cultured in the presence of 10 ng/ml RANKL (R&D Systems) and 10 ng/ml M-CSF for an additional 4 days to generate mature osteoclasts. If cells were cultured on bone slices, then an additional 2 days were needed.

**Design and construction of short hairpin ribonucleic acid (shRNA)**

Using the Dharmacon siDESIGN Centre (<http://www.dharmacon.com>) [2], we generated shRNA that would target Atp6v1c1. As a control vector, we used AAV-H1-shRNA-luc-YFP (gift from Dr. Sonoko Ogawa), which contains a luciferase-specific shRNA and a yellow fluorescent protein (YFP) cassette[3]. AAV-H1 contains a human Pol III H1 promoter for expression of shRNA as well as an independent green fluorescent protein (EGFP) expression cassette [4]. We cloned the H1 promoter shRNA expression cassette into the AAV construct as described [4-6]. The following shRNA oligonucleotides were annealed and cloned downstream of the H1 promoter of AAV-H1 into BglII and HindIII sites to produce AAV-H1-shRNA-Atp6v1c1: 5’-GATCCCC-TTCGTGACTTCCAGTATAA-**TTCAAGAGA**-TTATACTGGAAGTCACGAA-TTTTTGGAAT-3’ Nucleotides specific for targeting *Atp6v1c1* are underlined. The bold type signifies the 9-base pair hairpin spacer.

**AAV RNAi viral production and purification**

We used the AAV pHelper-Free System (AAV Helper-Free System, Stratagene) for viral production, which was accomplished using a triple-transfection, helper-free method, and purified with a modified version of a published protocol [5]. Briefly, HEK 293 cells were cultured in 150 × 25 mm cell culture dishes and transfected with pAAV-shRNA, pHelper and pAAV-RC plasmids (Stratagene) using a standard calcium phosphate method. Cells were collected after 60-72 hours and lysed via shaking with chloroform at 37^o^C for 1 hour. Sodium chloride was then added and shaken at room temperature for 30 minutes. The stock was spun at 12,000 RPM for 15 minutes and the supernatant was collected and cooled on ice for 1 hour with PEG8000. The solution was spun at 11,000 RPM for 15 minutes, and the pellet was treated with DNase and RNase. After the addition of chloroform and a five minute centrifugation at 12,000 RPM, the purified virus was in the aqueous phase at viral particle numbers of approximately 1 x 10^10^/ml. The AAV particle titer was determined using the AAV Quantitation Titer Kit (Cell Biolabs, Inc). To confirm the effect of silencing, we examined the expression of Atp6v1c1 in osteoclasts using qRT-PCR, Western blot, and immunofluorescence techniques. Luciferase expression vector AAV-Luc was purchased from North Carolina University. The AAV-luc was injected into the right side of the lower jaw and 14 or 35 days later luciferase expression was measured by an IVIS Imaging System 100 Series (Xenogen Corporation, Alameda, CA) as previously described [7].

**Acridine orange staining**

In present study, all of Acid production from different groups was determined by using acridine orange as described previously [8]. Osteoclasts that had been transduced with viral vectors after 1 day of RANKL/M-CSF stimulation and were incubated in α-MEM containing 5 ug/ml of acridine orange (Sigma) for 15 minutes at 37°C, and then washed and chased for 10 minutes in fresh media. The cells were observed under a fluorescence microscope with a 490 nm excitation filter and a 525 nm arrest filter. The experiment was performed in duplicate on three independent occasions in a 24-well plate.

***In vitro* bone resorption assays**

The extent of bone resorption in present study was assessed by following the protocol as described previously [9]. MBM cells were cultured on bovine cortical bone slices in 24-well plates and transduced with viral vectors after 1 day of RANKL/M-CSF stimulation. The bone slices were harvested after 6 days culture and the culture media was collected. Cells adhering to the bone slices were subsequently removed with 0.25M ammonium hydroxide and mechanical agitation. Bone slices were subjected to scanning electron microscopy (SEM) using a Philips 515 SEM (Department of Materials Science and Engineering, UAB). We also assessed *In vitro* bone resorption using wheat germ agglutinin (WGA) to stain exposed bone matrix proteins as described. The assays were performed in triplicate. The data were quantified by measuring the percentage of the areas resorbed in three random resorption sites, as determined using ImageJ analysis software.

**Western blotting analysis**

Western blotting was performed as previously outlined and visualized and quantified using a Fluor-S Multi-Imager with Multi-Analyst software (Bio-Rad) [10, 11]. A anti-Atp6v1c1 antibody (H-300) (Santa Cruz, CA) was used at a 1:1000 dilution, with goat anti-rabbit IgG-HRP (Cell signaling) used at a 1:5000 dilution to visualize the reaction.

**Reference List**

[1] Kelly KA, Tanaka S, Baron R, Gimble JM. Murine bone marrow stromally derived BMS2 adipocytes support differentiation and function of osteoclast-like cells in vitro. Endocrinology 1998;139: 2092-2101.

[2] Feng SM, Deng LF, Chen W, Shao JZ, Xu GL, Li YP. Atp6v1c1 is an essential component of the osteoclast proton pump and in F-actin ring formation in osteoclasts. Biochemical Journal 2009;417: 195-203.

[3] Alexander B, Warner-Schmidt J, Eriksson TM, Tamminga C, Arango-Lievano M, Ghose S, Vernov M, Stavarache M, Musatov S, Flajolet M, Svenningsson P, Greengard P, Kaplitt MG. Reversal of Depressed Behaviors in Mice by p11 Gene Therapy in the Nucleus Accumbens (vol 2, 62er4, 2010). Science Translational Medicine 2010;2.

[4] Musatov S, Chen W, Pfaff DW, Kaplitt MG, Ogawa S. RNAi-mediated silencing of estrogen receptor in the ventromedial nucleus of hypothalamus abolishes female sexual behaviors. Proceedings of the National Academy of Sciences of the United States of America 2006;103: 10456-10460.

[5] Hommel JD, Sears RM, Georgescu D, Simmons DL, DiLeone RJ. Local gene knockdown in the brain using viral-mediated RNA interference. Nature Medicine 2003;9: 1539-1544.

[6] Tomar RS, Matta H, Chaudhary PM. Use of adeno-associated viral vector for delivery of small interfering RNA. Oncogene 2003;22: 5712-5715.

[7] Tu QS, Zhang J, Fix A, Brewer E, Li YP, Zhang ZY, Chen J. Targeted Overexpression of BSP in Osteoclasts Promotes Bone Metastasis of Breast Cancer Cells. Journal of Cellular Physiology 2009;218: 135-145.

[8] Li YP, Chen W, Liang YQ, Li E, Stashenko P. Atp6i-deficient mice exhibit severe osteopetrosis due to loss of osteoclast-mediated extracellular acidification. Nature Genetics 1999;23: 447-451.

[9] Jules J, Shi ZQ, Liu JZ, Xu DR, Wang SQ, Feng X. Receptor Activator of NF-kappa B (RANK) Cytoplasmic IVVY535-538 Motif Plays an Essential Role in Tumor Necrosis Factor-alpha (TNF)-mediated Osteoclastogenesis. Journal of Biological Chemistry 2010;285: 37427-37435.

[10] Yang SY, Chen W, Stashenko P, Li YP. Specificity of RGS10A as a key component in the RANKL signaling mechanism for osteoclast differentiation. Journal of Cell Science 2007;120: 3362-3371.

[11] Yang SY, Wei DY, Wang D, Phimphilai M, Krebsbach PH, Franceschi RT. In vitro and in vivo synergistic interactions between the Runx2/Cbfa1 transcription factor and bone morphogenetic protein-2 in stimulating osteoblast differentiation. Journal of Bone and Mineral Research 2003;18: 705-715.
