# Supplementary material for: Targeting Atp6v1c1 Prevents Inflammation and Bone Erosion Caused by Periodontitis and Reveals Its Critical Function in Osteoimmunology
Source: PLoS One. 2015 Aug 14;10(8):e0134903. doi: 10.1371/journal.pone.0134903 (PMC4537256; doi:10.1371/journal.pone.0134903)
Supplement: S1 Table — (DOCX) [file pone.0134903.s002.docx]

**S1 Table.**qRT-PCR probe Numbers

| **Gene** | **Applied Biosystems Assay ID** |
| --- | --- |
| *tumor necrosis factor a (TNFα)* | Mm00443258_m1 |
| *osteoprotegerin (OPG)* | Mm01205928_m1 |
| *Cathepsin K (Ctsk)* | Mm00484039_m1 |
| Tumor necrosis factor (ligand) superfamily, member 11(RANKL) | Mm00441906_m1 |
| *Interleukin 6 (interferon, beta 2)(IL-6)* | Mm00446190_m1 |
| *Interleukin 17 receptor A (IL-17A)* | Mm00439618_m1 |

In this step, cDNA fragments were amplified by TaqMan® Fast Advanced Master Mix (Applied Biosystems). Fluorescence from each TaqMan probe was detected using the Step-One real-time PCR system (Applied Biosystems). The mRNA expression level of the housekeeping gene hypoxanthine-guanine phosphoribosyl transferase (*Hprt*) was used as an endogenous control and enabled calculation of specific mRNA expression levels as a ratio of *Hprt (deltaCT)*. PCR was performed under standard conditions and repeated at least three times.
